# Targeting drug efflux and DNA repair enhances inotuzumab ozogamicin activity in IO-resistant B-ALL cell lines

**DOI:** 10.1007/s12185-026-04221-2

**Published:** 2026-05-11

**Authors:** Naoko Ida, Miyuki Okura, Saki Tanaka, Naoko Hosono, Takahiro Yamauchi

**Affiliations:** 1 Department of Hematology and Oncology, University of Fukui, 23-3, Shimoaizuki, Matsuoka, Eiheiji, Fukui, 910-1193 Japan; 2 Department of Diabetes, Endocrinology, and Hematology, DMU Saitama Medical Center, 2-1-50 Minami-Koshigaya, Koshigaya-Shi, Saitama, 343-8555 Japan; 3 Department of Blood Transfusion, University of Fukui Hospital, 23-3, Shimoaizuki, Matsuoka, Eiheiji, Fukui, 910-1193 Japan

**Keywords:** Acute lymphoblastic leukemia, Inotuzumab ozogamicin, Drug efflux, DNA damage repair

## Abstract

Inotuzumab ozogamicin (IO), an anti-CD22 antibody conjugated with calicheamicin, is highly effective against relapsed/refractory B-cell acute lymphoblastic leukemia (B-ALL). However, acquired resistance limits the long-term efficacy of IO. To elucidate the underlying mechanisms of IO resistance and explore strategies to overcome it, six IO-resistant B-ALL sublines were newly established. Although CD22 expression was preserved in all resistant sublines, more than 80-fold increases in 50% inhibitory concentration (IC50) were observed, accompanied by acquired resistance to IO-induced apoptosis. Microarray analysis revealed upregulation of ABCB1, which was further confirmed by overexpression of the encoded protein, the drug efflux pump P-glycoprotein (P-gp). P-gp inhibition restored IO sensitivity by inhibiting calicheamicin efflux, a key resistance mechanism. Concurrently, poly (ADP-ribose) polymerase (PARP) inhibition of DNA damage repair enhanced IO cytotoxicity. Notably, in the triplet combination of IO with P-gp and PARP inhibitors, PARP inhibition of the repair of DNA damage caused by calicheamicin accumulation following P-gp inhibition markedly enhanced the antitumor effects of IO. This approach may offer a novel and effective therapeutic strategy for IO-resistant B-ALL.

## Introduction

Acute lymphoblastic leukemia (ALL) is a hematological malignancy characterized by the inhibition of lymphoid progenitor cell differentiation and aberrant proliferation [1–4]. While the prognosis has been greatly improved for pediatric patients with ALL [5], outcomes for adult ALL remain poor, and improvements in therapeutic strategies are sorely needed [6]. Once ALL relapses, salvage treatments using conventional anti-leukemic drugs have generally been unsatisfactory, with a complete response rate of 30–50% [7]. Median overall survival for relapsed/refractory ALL treated with conventional chemotherapy is less than 1 year [7]. Hematopoietic stem cell transplantation is the only curative modality for relapsed/refractory adult ALL [8, 9]. Further, achievement of complete response by salvage treatment before transplantation is a prerequisite to successful transplantation and long-term survival.

Recently, inotuzumab ozogamicin (IO), blinatumomab, and chimeric antigen receptor-T cell therapy (CAR-T) have been introduced [10–15], and those new options have greatly improved remission rates for relapsed/refractory ALL. Among these, IO is an antibody–drug conjugate (ADC) for the treatment of B-cell ALL (B-ALL) [16–19]. IO consists of a humanized immunoglobulin (Ig)G4 monoclonal antibody targeting CD22 antigen, which is commonly expressed on B-lymphoblasts, conjugated to the cytotoxic agent calicheamicin via a linker [19, 20]. IO binds to the CD22 antigen on the leukemic cell surface and is then internalized into the cell. Lysosomal enzymes cut the linker; then, free calicheamicin enters the nucleus and binds to the minor groove of DNA. Calicheamicin induces single- and double-stranded breaks in DNA, thereby triggering apoptosis [20]. The cytotoxic effect of IO thus depends on the induction of DNA strand breaks. In the INO-VATE clinical trial, IO demonstrated a significantly higher complete remission rate compared to standard chemotherapy; however, improvements in progression-free and overall survival were limited [21]. This limited efficacy is largely attributed to the acquisition of IO resistance, and the underlying mechanisms of this resistance remain unclear. The aim of the present study was to establish novel IO-resistant ALL cell lines through continuous in vitro drug exposure and to comprehensively investigate the mechanisms underlying resistance. In our previous study, inhibition of DNA damage repair pathways augmented the cytotoxic effects of IO [22]. Based on this finding, we further evaluated whether this approach could overcome resistance in newly established IO-resistant cell lines.

## Materials and methods

### Cell culture and establishment of IO-resistant cell lines

The Reh Philadelphia-negative B-ALL cell line (cat. no. CRL-8286) was purchased from the American Type Culture Collection (Manassas, VA, USA). Reh cells were cultured in RPMI 1640 medium supplemented with 10% fetal bovine serum (Sigma-Aldrich, St. Louis, MO, USA) at 37 °C in a humidified atmosphere containing 5% CO_2_. To establish IO-resistant cell lines, Reh cells were initially exposed to IO at a concentration equivalent to 1% of the 50% inhibitory concentration (IC_50_); then, the concentration of IO was incrementally increased by 0.1 ng/mL, contingent on cell proliferation. Cells were cultured until reaching a density of 1 × 10^6^ cells/mL before passage. IC_50_ values were periodically monitored throughout passage with IO exposure. Once the IC_50_ of the bulk cultured cells was over 100-fold higher than that of the parental cells, single-cell cloning was performed using the limiting dilution method [23].

### Chemicals and reagents

IO was a gift from Pfizer (New York, NY, USA). PSC-833 (cat. no. ab145870) was purchased from Abcam (Cambridge, UK). Olaparib (cat. no. Ku-0059436), talazoparib (cat. no. LT-673), etoposide (cat. no. S1225), and cytarabine (cat. no. S1648) were purchased from Selleck Biotech (Houston, TX, USA). IO was dissolved in water for injection (7131400A2145; FUSO Pharmaceutical Industries, Tokyo, Japan). All other small-molecule inhibitors were dissolved in dimethyl sulfoxide (DMSO) (cat. no. 046—21,981; FUJIFILM Wako Pure Chemical Corporation, Osaka, Japan) to prepare stock solutions.

### Cell growth inhibition assay

To determine the growth-inhibitory effects of drugs, parental and IO-resistant cells were treated with serially diluted concentrations of drugs, either alone or in combination, for 48 h [24]. Cell viability was quantified using Cell Counting Kit-8 (CCK-8) (cat. no. CK04; Dojindo, Kumamoto, Japan) based on the water-soluble tetrazolium salt assay according to the protocol from the manufacturer. Absorbance at a wavelength of 450 nm was measured using a SPECTRA MAX 250 microplate reader (Molecular Devices, CA, USA). IC_50_ values were calculated from the resulting dose–response curves.

### Cell surface CD22 positivity

Cells were stained with a Fluorescein isothiocyanate (FITC)-conjugated anti-CD22 antibody (clone SJ10.1H1, cat. no. IM0779U; Beckman Coulter, Brea, CA, USA). CD22 positivity was determined using a BD FACSCanto II flow cytometer (BD Biosciences, Franklin Lakes, NJ, USA).

### Annexin V binding assay

Drug-induced apoptosis was quantified by annexin V and propidium iodide (PI) co-staining. After drug treatment, cells were collected and stained using Annexin V-FITC Apoptosis Detection Kit I (cat. no. 11 858 777 001; Roche Diagnostics, Mannheim, Germany). Samples were analyzed using a flow cytometer and FACSDiva software (version 6.1.3; Becton Dickinson, New Jersey, USA).

### DNA microarray analysis

To investigate differential gene expression profiles between IO-resistant cell lines and their parental cells, microarray analysis was performed. Total RNA was extracted from 1 × 10^5^ cells using the RNeasy Mini Kit (cat. no. 74104; QIAGEN, Hilden, Germany). The quality and integrity of the RNA were assessed using an Agilent 2100 Bioanalyzer (Agilent Technologies, Santa Clara, CA, USA). Only samples with RNA Integrity Number ≥ 8.0 were used for subsequent analysis. Biotin-labeled targets were prepared from total RNA samples. Complementary RNA (cRNA) was synthesized then used to generate single-stranded complementary DNA (ss-cDNA), which was subsequently fragmented and labeled using the GeneChip™ WT PLUS Reagent Kit (cat. no. 902280; Thermo Fisher Scientific, Waltham, MA, USA). The labeled ss-cDNA was hybridized to Clariom S Assay, Human Array (cat. no. 902927; Thermo Fisher Scientific), followed by staining using a GeneChip™ Fluidics Station 450 and scanning with a GeneChip™ Scanner 3000 7G (Thermo Fisher Scientific). Data were analyzed using Transcriptome Analysis Console software (version 4.0; Thermo Fisher Scientific).

### Western blotting analysis

Protein expression levels of P-gp were analyzed by Western blotting. Cells were pelleted, washed three times with phosphate-buffered saline (PBS), then lysed with 100 μL of RIPA buffer (cat. no. 4987481492248; FUJIFILM Wako Pure Chemical Corporation, Osaka, Japan) on ice for 10 min. After centrifugation at 14,500 rpm for 15 min, the supernatant containing the protein extract was collected. After protein transfer, membranes were incubated overnight at 4 °C with the following primary antibodies: rabbit polyclonal anti-P-gp antibody (1:500 dilution; cat. no. 22336—1-AP; Proteintech Group, Rosemont, IL, USA) and a mouse monoclonal anti-Glyceraldehyde-3-phosphate dehydrogenase (GAPDH) antibody (1:1000 dilution; cat. no. MAB374; Merck KGaA, Darmstadt, Germany). GAPDH was used as a loading control. After washing with Tris-buffered saline tween (TBS-T), the membrane was incubated with horseradish peroxidase-conjugated secondary antibodies (1:10,000 dilution) for 60 min at room temperature. The intensity of each band was quantified by densitometry.

### Multidrug resistance assay

The function of P-gp was assessed using a Multidrug Resistance Assay Kit (cat. no. 600370; Cayman Chemical, Ann Arbor, MI, USA). Cells (2 × 10^5^ cells/mL) were pre-incubated for 30 min at 37 °C with PSC-833 (0.1 µM), positive controls (cyclosporin A or verapamil, supplied with the kit), and vehicle alone (negative control). After pre-incubation, the fluorescent P-gp substrate Calcein AM was added to all samples, and then, the cells were incubated for 30 min. The reaction was stopped by centrifugation at 400 × *g* for 5 min. The cell pellet was resuspended in culture medium containing PI to exclude non-viable cells. Intracellular fluorescence of Calcein AM was quantified in the viable (PI-negative) cell population using a BD FACSCanto™ II flow cytometer (Becton Dickinson, New Jersey, USA). P-gp function was evaluated based on Calcein AM retention.

### Alkaline comet assay

DNA strand breaks were quantified using the alkaline OxiSelect™ Comet Assay Kit (cat. no. STA-351; Cell Biolabs, San Diego, CA, USA). IO-resistant cells (1 × 10^5^ cells/mL) were incubated for 1 or 6 h at 37 °C with IO (2 ng/mL) either alone or in combination with PSC-833 (0.1 µM), olaparib (1 µM), or talazoparib (100 nM). After treatment, cells were mixed with low-melting-point agarose and layered onto OxiSelect™ Comet Slides. Embedded cells were then sequentially lysed and subjected to DNA unwinding in alkaline solution prior to electrophoresis. Slides were washed, fixed in 70% ethanol for 5 min at room temperature, and air-dried. DNA was stained with Vista Green DNA dye, and comets were visualized using a BX50F epifluorescence microscope (Olympus Corporation, Tokyo, Japan). Image analysis was performed using Comet Assay IV software (version 4.0; Oxford Instruments, Abingdon, UK) to calculate the Olive tail moment. For each experimental condition, a minimum of 50 randomly selected cells were analyzed per replicate [22].

#### Primary B-ALL patient samples and Ex Vivo drug sensitivity assay

Primary leukemia cells were obtained from the peripheral blood of two patients with B-ALL (one Philadelphia chromosome-negative and one Philadelphia chromosome-positive) during routine clinical care. The study was conducted in accordance with the Declaration of Helsinki and was approved by the Institutional Review Board of The Research Ethics Committee of University of Fukui. Written informed consent was obtained from all patients. Mononuclear cells were isolated by density gradient centrifugation using Ficoll (FUJIFILM Wako, Japan) and cryopreserved in Cell Banker (TaKaRa Bio, Japan) until use. For the ex vivo drug sensitivity assay, thawed cells were seeded at a density of 1 × 10^5^ cells/mL. Ph-negative sample was treated with IO (30 ng/mL), while Ph-positive sample was treated with IO (50 ng/mL), either alone or in combination with Olaparib (10 µM) or Talazoparib (1 nM). After 48 h of incubation, cell viability was assessed using the trypan blue exclusion assay. Cells excluding trypan blue were considered viable. The percentage of cell death was calculated using the following formula: cell death (%) = [number of dead cells / (number of dead cells + number of viable cells)] × 100.

## Results

### Characteristics of newly established IO-resistant sublines

Six IO-resistant sublines were successfully established (Reh IO-R A, B, C, D, E, and F). The IC_50_ of IO for the parental Reh cell line was 5.3 ng/mL. In the six resistant sublines, IC_50_ values were more than 80-fold higher than that of the parental cell line. The most resistant subline, Reh-IO-R A, exhibited approximately 144-fold higher resistance compared with the parental cells (Table 1). To characterize the acquired resistance to IO, cell surface CD22 expression was assessed. Parental cells exhibited a high level of CD22 expression (84.0% positive). Among the six resistant sublines, CD22 expression levels ranged from 61.7% (Reh-IO-R C) to 87.6% (Reh-IO-R F), although complete loss of antigen was not observed in any sublines (Table 1).

**Table 1:** CD22 expression and IC_50_ values of IO in six IO-resistant sublines

	CD22 positivity [%]	IC_50_ of IO [ng/mL]
Reh	84	5.3
Reh-IO-R A	69.2	762.3
Reh-IO-R B	66.9	714.9
Reh-IO-R C	61.7	582.5
Reh-IO-R D	85.7	474.6
Reh-IO-R E	83.9	541.1
Reh-IO-R F	87.6	606.6

CD22 expression was analyzed by FACS, and IC_50_ values of IO were determined using WST assay. CD, cluster of differentiation; FACS, fluorescence-activated cell sorting; IC_50_, half maximal inhibitory concentration; IO, inotuzumab ozogamicin; WST, water-soluble tetrazolium

### IO-resistant cells were refractory to IO-induced apoptosis

**Fig. 1:**
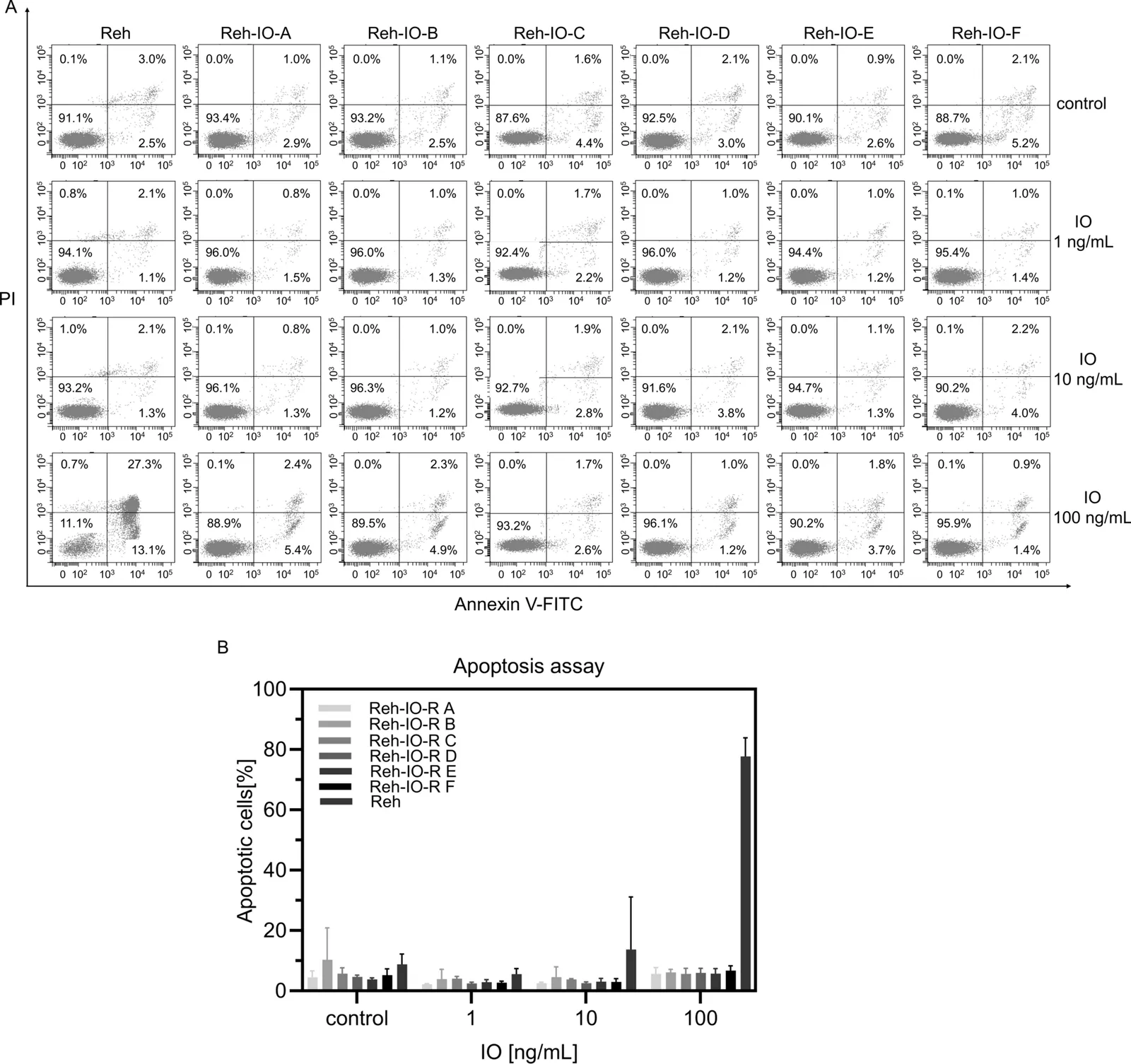
Induction of apoptosis by IO in IO-resistant sublines and the parental cell line. Six IO-resistant sublines and the parental cell line were incubated with or without 1, 10, or 100 ng/mL of IO for 48 h, followed by Annexin V/PI staining and flow cytometric analysis. **A** In the representative dot plots, the lower left quadrant indicates live cells, the lower right quadrant indicates early apoptotic cells, the upper right quadrant indicates late apoptotic cells, and the upper left quadrant indicates necrotic cells. **B** The histogram shows the percentage of apoptotic cells. IO, inotuzumab ozogamicin

To determine whether the resistance involved impaired apoptosis, induction of apoptosis was assessed in parental and IO-resistant cells after 48 h of IO exposure using the annexin V binding assay. As shown in Fig. 1, IO induced a dose-dependent increase in apoptotic cells in parental cells, whereas apoptosis was barely induced in any of the six IO-resistant sublines, even at 100 ng/mL IO.

### Drug transporter *ABCB1* was upregulated in IO-resistant sublines

**Fig. 2:**
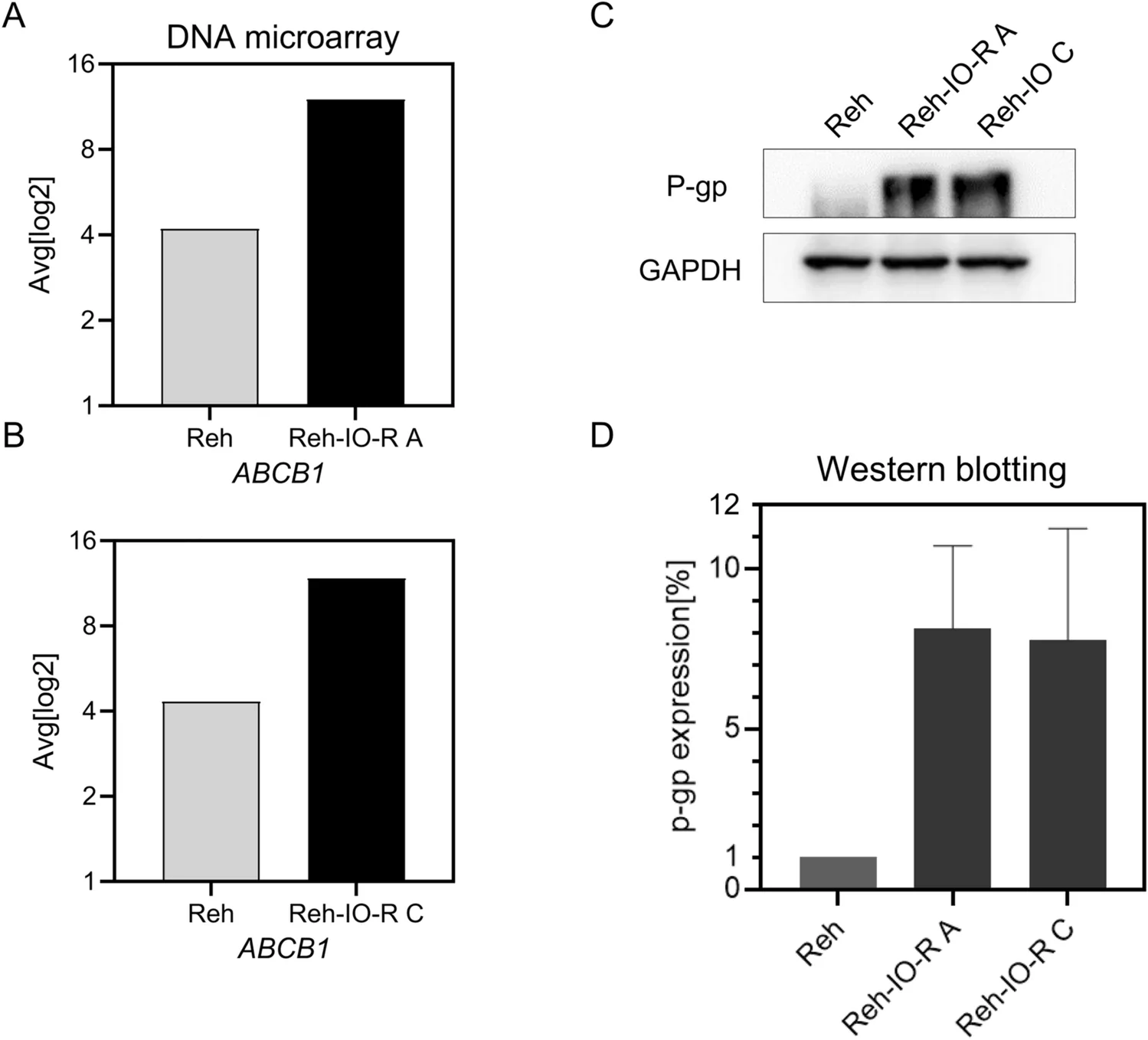
ABCB1 expression in IO-resistant and parental cell lines. DNA microarray analysis was performed to assess differential gene expression. The average expression signal of each resistant subline (**A**: Reh-IO-R A, **B**: Reh-IO-R C) was compared with that of the parental line. **C** P-gp protein expression was analyzed by Western blotting. **D** Densitometric analysis of P-gp expression normalized to GAPDH

To elucidate potential molecular mechanisms underlying IO resistance, gene expression microarray analysis was performed. Parental cells were compared with two representative IO-resistant sublines: Reh-IO-R A, exhibiting the highest IC_50_, and Reh-IO-R C, characterized by the lowest CD22 expression. As shown in Fig. 2A and B, *ABCB1* transcripts were markedly upregulated in both resistant sublines relative to parental cells. Consistently, Western blotting analysis demonstrated overexpression of P-gp coded by *ABCB1* in both Reh-IO-R A and C and was validated by densitometric quantification (Fig. 2C, D). In contrast, expression levels of apoptosis-related molecules and DNA repair factors were unchanged in IO-resistant sublines (Table 2).

**Table 2:** Gene expression profiles of parental and IO-resistant B-ALL cell lines

Gene	Parental cells (log2)	IO-resistant cells (log2)
BCL-2	14.2	13.4
BAX	9.3	9.3
Bcl-xL	11	10.8
MCL-1	13.5	13.4
TP53	14.5	14.6
ERCC1	12	13
PARP1	13.1	13.2
PARP2	11.3	11.2
PARP3	7	6.7
BRCA1	10.8	10.1
BRCA2	11.4	11.3
ABCB1	4	11.7
ABCB2	4.9	4.9
MDR	9.7	9.4

Gene expression levels were determined by microarray analysis as described in Materials and Methods. Values are presented as log2 expression levels. IO, inotuzumab ozogamicin; B-ALL, B-cell acute lymphoblastic leukemia

### Inhibition of P-gp drug efflux function enhanced IO sensitivity

**Fig. 3:**
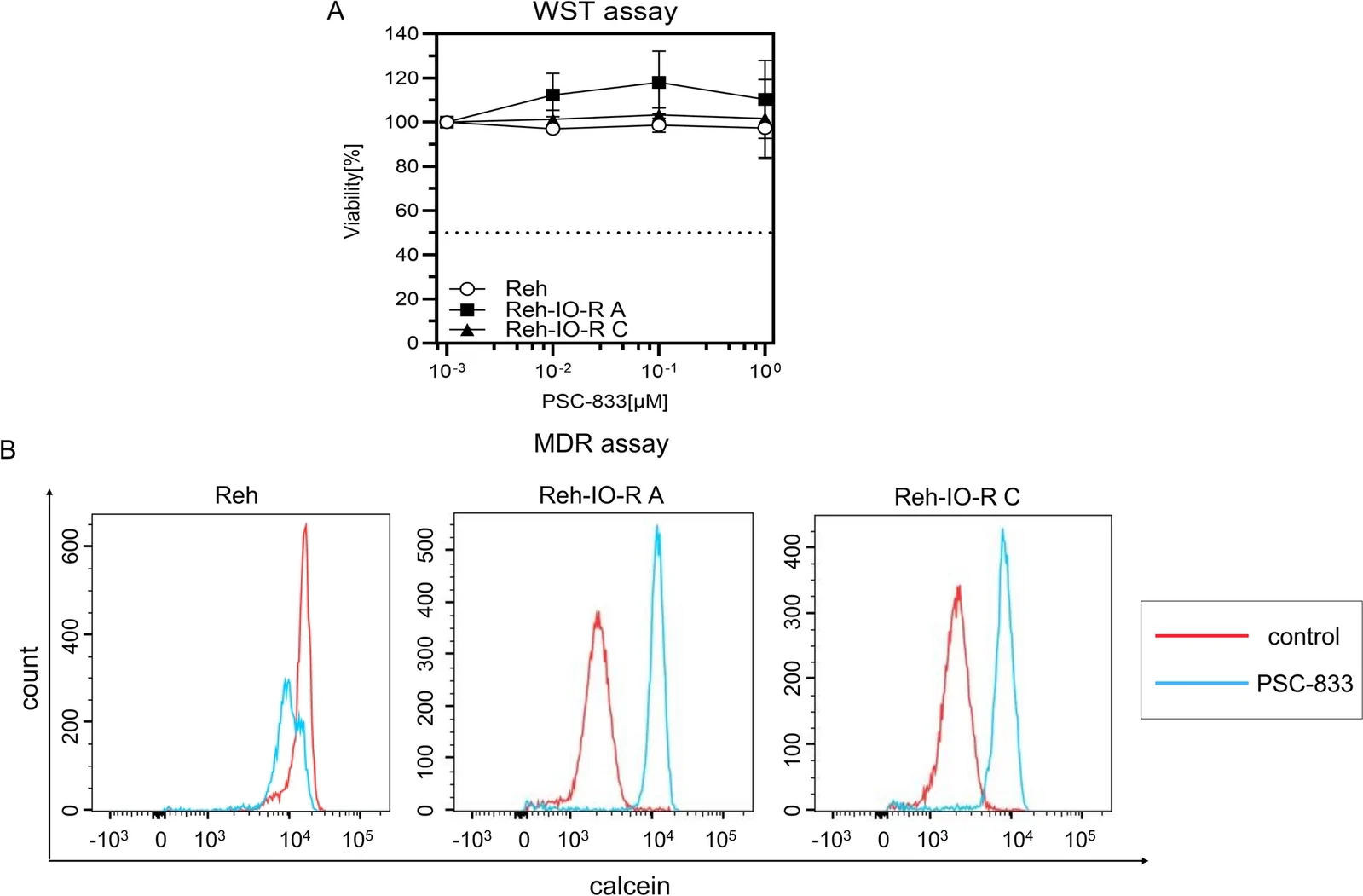
Inhibitory effect of PSC-833, a P-gp inhibitor, on cell proliferation and drug efflux function. **A** Cell proliferation was assessed by WST assay in IO-resistant and parental cell lines treated with PSC-833. **B** MDR assay was performed using flow cytometry to evaluate the effect of PSC-833 on drug efflux. P-gp, P-glycoprotein; WST, water-soluble tetrazolium; IO, inotuzumab ozogamicin; MDR, multidrug resistance

The intrinsic cytotoxicity of PSC-833 was evaluated, and no cytotoxic effects were observed even at high concentrations (Fig. 3A). A multidrug-resistant assay was performed to confirm functional inhibition of P-gp by PSC-833 (Fig. 3B). Resistant sublines overexpressing P-gp exhibited low intracellular fluorescence, consistent with active efflux of the Calcein AM substrate. In contrast, cells treated with PSC-833 (0.1 µM) retained intracellular fluorescence comparable to that of cells treated with positive controls (cyclosporine A or verapamil), indicating inhibition of P-gp-mediated efflux of the Calcein AM substrate. To evaluate cross-resistance, we determined sensitivity to other cytotoxic agents. The IO-resistant cell lines exhibited moderate cross-resistance to etoposide, known to be a P-gp substrate. In contrast, IC_50_ values for cytarabine were comparable between the IO-resistant cell lines and the parental cells (Table 3)

**Table 3:** Cross-resistance of IO-resistant B-ALL cell lines to etoposide and cytarabine

Cell lines	IC_50_ of etoposide [nM]	IC_50_ of cytarabine [nM]
Reh	92.2	78.2
A	252.6	39.7
C	283.3	40.1

IC_50_ values of etoposide (a P-gp substrate) and cytarabine (a non–P-gp substrate) were determined using the WST assay in IO-resistant cell lines to assess cross-resistance associated with P-gp–mediated drug efflux. IC₅₀, half-maximal inhibitory concentration; WST, water-soluble tetrazolium

.

**Fig. 4:**
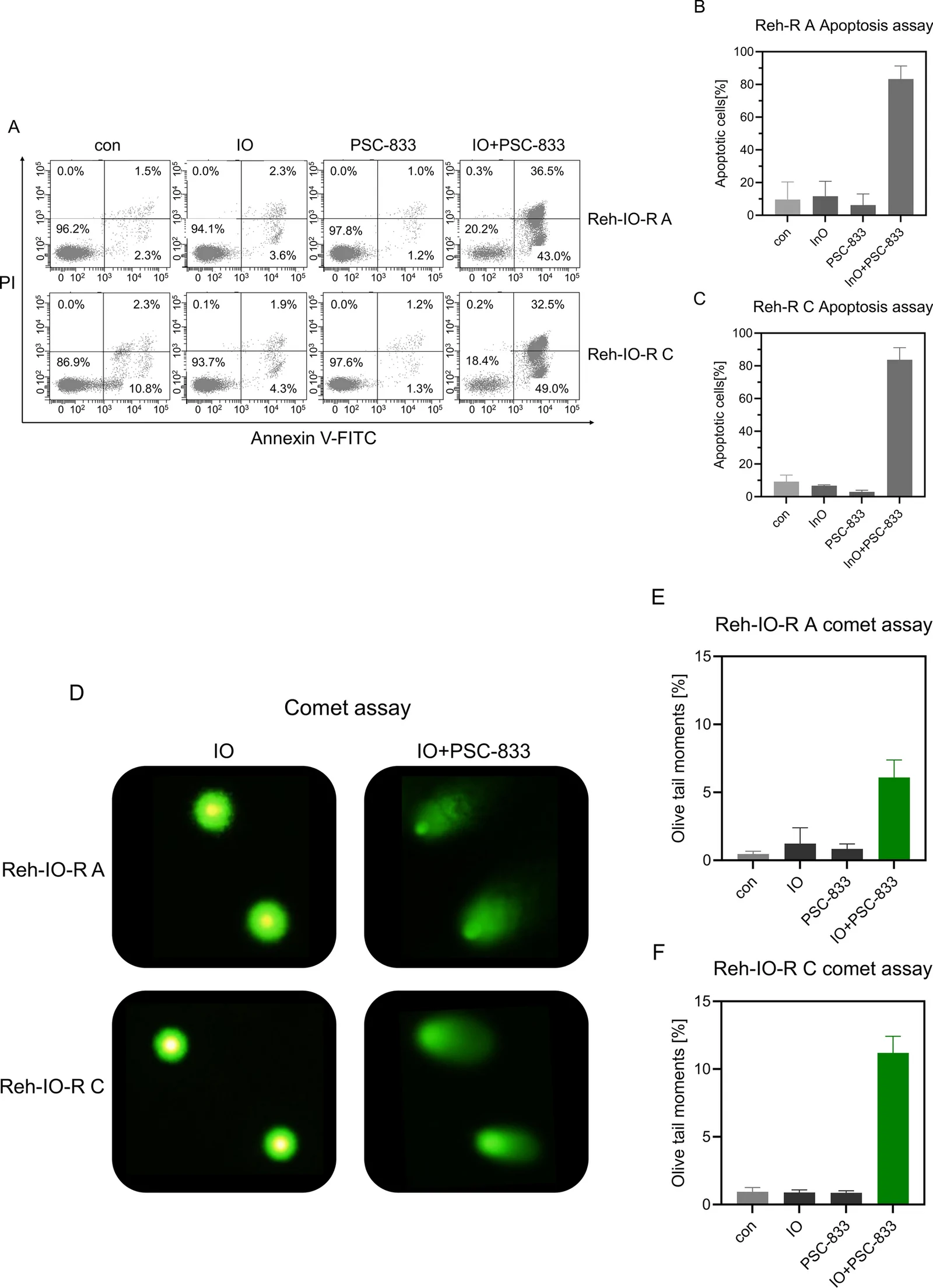
The combination effect of IO and PSC-833. Apoptosis was evaluated by Annexin V/PI staining. Cells were incubated with 100 ng/mL IO, 0.1 μM PSC-833, or the combination for 48 h, followed by Annexin V/PI co-staining. **A** In the representative dot plots, the lower left quadrant indicates live cells, the upper left quadrant indicates necrotic cells, and the lower and upper right quadrants (Annexin V⁺) indicate early and late apoptotic cells, respectively. **B**, **C** The bar graphs show the percentage of Annexin V–positive cells. Error bars indicate the standard deviation from at least three independent experiments. Alkaline comet assay was used to quantify the extent of DNA damage. Cells were incubated with 1000 ng/mL IO with or without 0.1 μM PSC-833 for 1 h, followed by comet assay. **D** Representative images of comets show DNA damage. **E**, **F** Olive tail moment was calculated as Tail DNA% × Tail Moment Length. Bar graphs show the olive tail moment values, with error bars indicating standard deviation from at least three independent experiments

Reh-IO-R A and C cells were exposed to IO alone or in combination with PSC-833. Combination treatment markedly enhanced IO sensitivity in Reh-IO-R A and C cell lines compared with IO alone (Table 4). Moreover, co-treatment with IO (100 ng/mL) and PSC-833 significantly increased the proportion of apoptotic cells in both resistant sublines relative to either agent alone (Fig. 4A–C). Inhibition of P-gp was hypothesized to increase DNA damage by inhibiting the efflux of the calicheamicin payload of IO. The Comet assay was therefore employed to quantify calicheamicin-induced DNA strand breaks. Neither IO nor PSC-833 alone induced significant DNA damage at 1 h, whereas combined treatment with IO and PSC-833 markedly increased DNA strand breaks, as evidenced by prominent comet tails (Fig. 4D). The Olive tail moment, a quantitative measure of DNA damage, was significantly elevated only in the combination treatment group in both resistant sublines (Fig. 4E, F).

**Table 4:** IC₅₀ values of IO alone or in combination with PSC-833 and/or PARP inhibitors in IO-resistant cell lines

Reh-IO-R sublines	IC_50_ of IO [ng/mL]					
	IO	IO + PSC	IO + ola	IO + tala	IO + PSC + ola	IO + PSC + tala
A	762.3	21.6	406.2	240.5	7.1	4.8
C	582.5	22	290.3	59.3	4	3.2

IO-resistant cell lines were treated with IO alone, IO plus the P-gp inhibitor PSC-833 (0.1 μM), IO plus PARP inhibitors (1 μM olaparib or 100 nM talazoparib), or the triple combination of IO, PSC-833, and PARP inhibitors for 48 h. The IC₅₀ values of IO were determined using the WST assay. IO, inotuzumab ozogamicin; P-gp, P-glycoprotein; IC₅₀, half-maximal inhibitory concentration; WST, water-soluble tetrazolium; Ola, olaparib; Tala, talazoparib

### Inhibition of DNA damage repair by poly(ADP-ribose) polymerase (PARP) inhibitors enhanced IO sensitivity

**Fig. 5:**
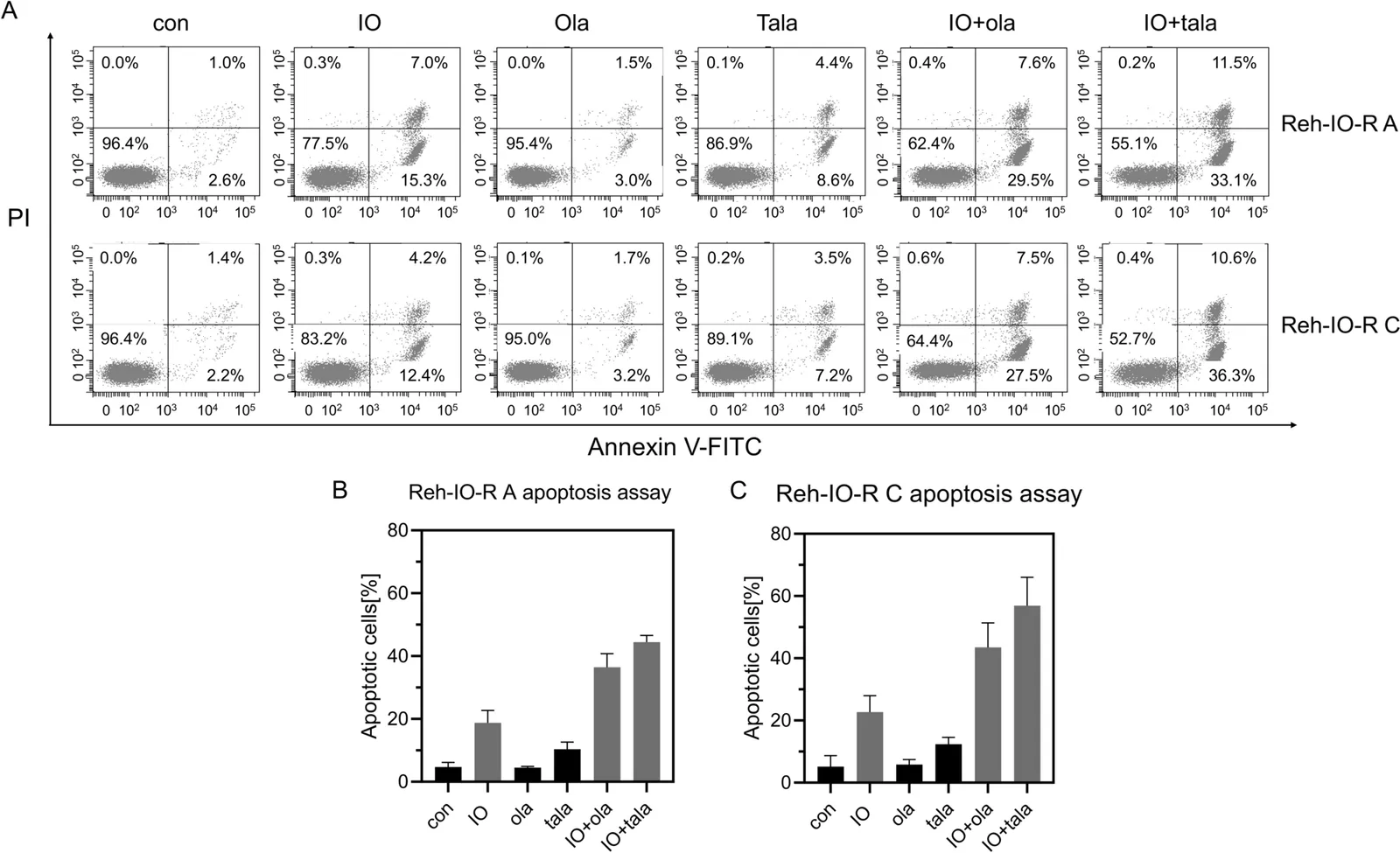
Induction of apoptosis by IO with olaparib or talazoparib. Cells were incubated with 1000 ng/mL IO, with or without 1 μM olaparib and 100 nM talazoparib, for 48 h, followed by Annexin V/PI co-staining. **A** In the representative dot plots, the lower left quadrant indicates live cells, the upper left quadrant indicates necrotic cells, and the lower and upper right quadrants (Annexin V⁺) indicate early and late apoptotic cells, respectively. **B**, **C** The bar graphs show the percentage of Annexin V–positive cells. Error bars indicate the standard deviation from at least three independent experiments

Based on our previous study, PARP inhibitors were hypothesized to inhibit the IO-induced DNA damage repair pathway, resulting in enhanced cytotoxicity of IO [22]. IO-resistant sublines A and C were treated with IO with or without non-cytotoxic concentrations of the PARP inhibitors olaparib (1 µM) or talazoparib (100 nM). Both PARP inhibitors significantly enhanced the cytotoxicity of IO in resistant cells (Table 3). Talazoparib enhanced the cytotoxicity of IO more potently than olaparib in both sublines. Moreover, a significant increase in the apoptotic cell population was observed by IO combined with either olaparib or talazoparib (Fig. 5A-C).

### Triple combination with P-gp inhibitor, PARP inhibitor, and IO reversed IO resistance

**Fig. 6:**
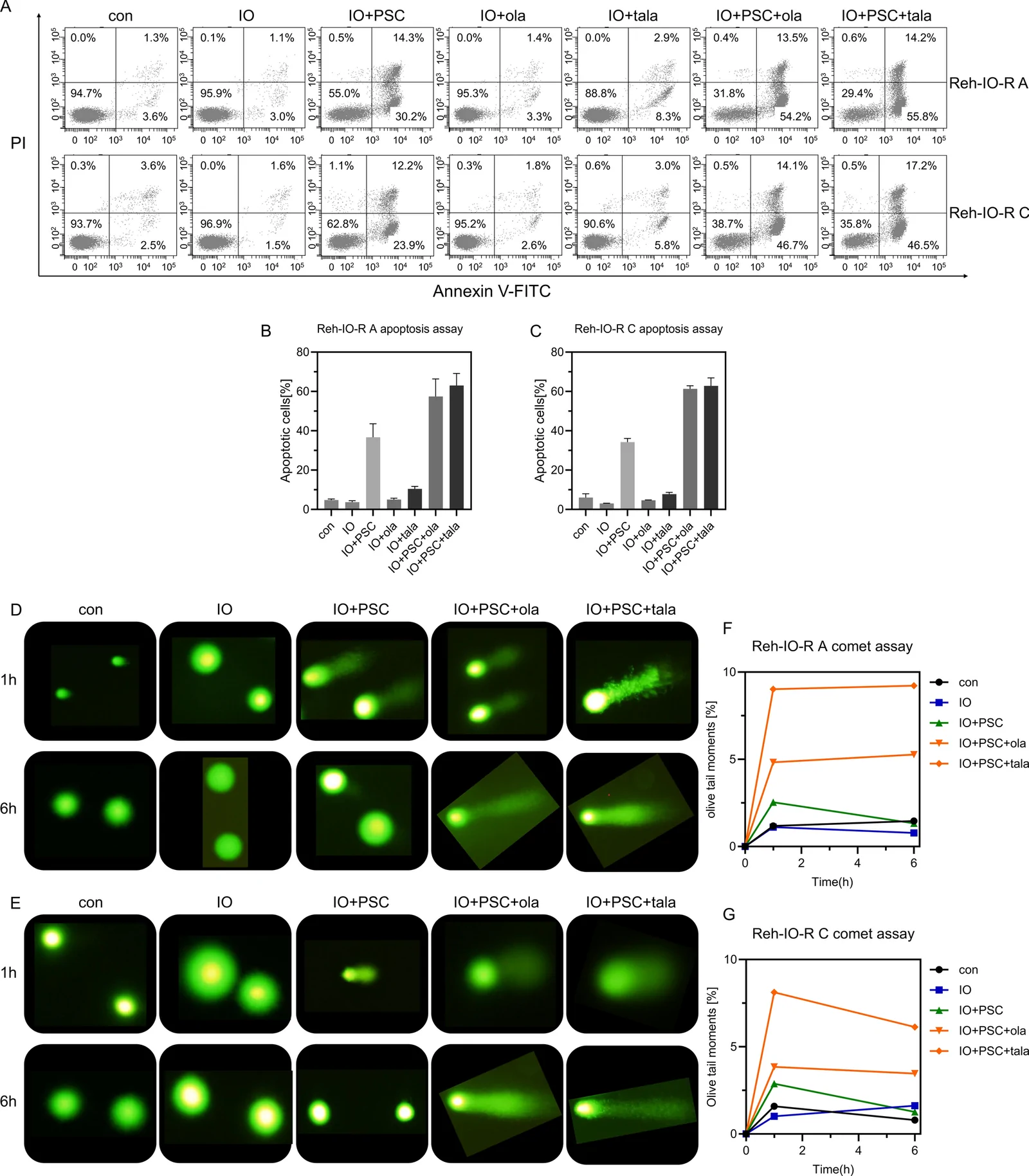
The triplet combination effect of IO, PSC-833, and olaparib or talazoparib. Cells were incubated with 10 μg/mL IO, with or without 0.1 μM PSC-833, 1 μM olaparib, and 100 nM talazoparib for 48 h, followed by Annexin V/PI co-staining. **A** In the representative dot plots, the lower left quadrant indicates live cells, the upper left quadrant indicates necrotic cells, and the lower and upper right quadrants (Annexin V⁺) indicate early and late apoptotic cells, respectively. **B**, **C** The bar graphs show the percentage of Annexin V–positive cells. Error bars indicate the standard deviation from at least three independent experiments. Alkaline comet assay was used to quantify DNA damage. Cells were incubated with 1,000 ng/mL IO with or without 0.1 μM PSC-833 or 1 μM olaparib plus 100 nM talazoparib for 1 h and 6 h. **D**, **E** Representative images of comets show DNA damage in Reh-IO-A (**D**) and Reh-IO-C (**E**) cells. **F**, **G** Olive tail moment (%) was calculated as Tail DNA% × Tail moment length. Line graphs show changes in olive tail moment over the indicated time points for Reh-IO-A (**F**) and Reh-IO-C (**G**) cells

The cytotoxicity of the triple combination of IO, P-gp inhibitor (PSC-833), and PARP inhibitor (olaparib or talazoparib) was evaluated in resistant cell lines. Notably, addition of both PSC-833 and the PARP inhibitor to IO reduced IC_50_ values in resistant sublines to levels comparable to those of parental cells (Table 3). The proportion of apoptotic cells was increased by double combinations of IO with either P-gp inhibitor or PARP inhibitor compared with IO alone and was further enhanced by the triple combination (Fig. 6A-C).

To elucidate the mechanism underlying the combination effect, the kinetics of DNA damage were assessed using the comet assay. Treatment with IO plus PSC-833 induced greater DNA damage at 1 h compared with IO alone. However, this damage was repaired by 6 h via intrinsic DNA repair pathways, resulting in levels comparable to those observed with IO alone or control. In contrast, the triplet combination not only induced greater DNA damage at 1 h but also achieved sustained damage through 6 h (Fig. 6D-G). These findings suggest that PSC-833 enhances intracellular retention of calicheamicin by inhibiting the drug efflux pump, while the PARP inhibitor abrogates the repair of calicheamicin-induced DNA damage, thereby leading to persistent DNA strand breaks.

### Ex vivo cytotoxicity of IO and PARP inhibitor combinations in primary B-ALL cells

**Fig. 7:**
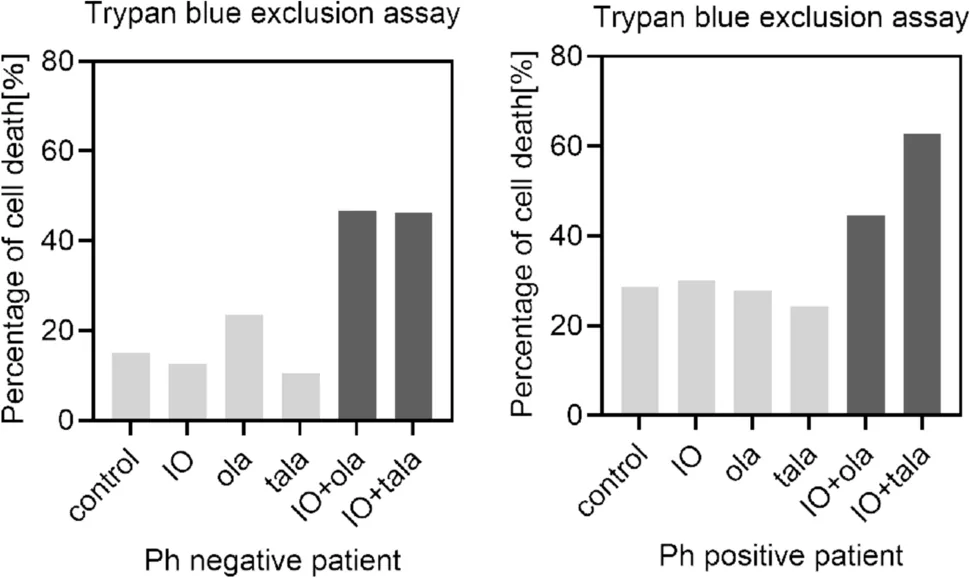
Ex vivo cytotoxicity of IO in combination with PARP inhibitors in primary B-ALL cells. Primary B-ALL cells from Ph-negative and Ph-positive patients were treated with IO alone or in combination with olaparib or talazoparib. Cell viability was assessed after 48 h using the trypan blue exclusion assay

We assessed the cytotoxic effects of IO combined with olaparib or talazoparib using primary B-ALL cells. In both Philadelphia chromosome (Ph)-negative and Ph-positive patient samples, the combination of IO and PARP inhibitors resulted in increased cell death compared with IO alone (Fig. 7).

## Discussion

Six IO-resistant sublines were established, all showing marked resistance to growth inhibition and apoptosis (Fig. 1). Two sublines, Reh-IO-R A and C, with the highest IC_50_ and lowest CD22 expression, respectively, were selected for further analysis (Table 1). Both exhibited upregulated ABCB1 mRNA and increased P-gp protein (Fig. 2). Addition of P-gp or PARP inhibitors further decreased the IC_50_ of IO and enhanced apoptosis (Figs. 4–5). Moreover, the triple combination of IO with P-gp and PARP inhibitors significantly increased IO cytotoxicity (Fig. 6).

The mechanisms of resistance to anticancer agents are generally multifactorial, including alterations in targets such as genetic mutations, amplification, or protein overexpression; intracellular drug degradation; enhanced repair of drug-induced DNA damage; and anti-apoptotic effects [24–26]. GO, an ADC similar to IO, consists of an anti-CD33 antibody conjugated to calicheamicin and is used for the treatment of relapsed/refractory acute myeloid leukemia [27, 28]. In our previous study, resistance mechanisms to GO were found to include decreased CD33 antigen expression, emergence of drug efflux pumps such as P-gp and multidrug resistance-associated protein 1, and enhanced DNA strand break repair [29, 30]. In addition, overexpression of the anti-apoptotic proteins Bcl-2 and Mcl-1 is reportedly associated with GO resistance [31–35].

Expression of drug efflux pumps represents one of the major mechanisms of resistance to anthracyclines [36, 37]. Calicheamicin is also a substrate of drug efflux pumps [38]. P-gp is an ATP-dependent drug efflux pump expressed on the cell surface, actively transporting substrate agents from the intra to the extracellular space by utilizing the energy derived from ATP hydrolysis [39] and affects the pharmacokinetics of various agents [40–42]. The antitumor effect of IO is ultimately mediated by DNA damage induced by its payload, calicheamicin. Takeshita et al. previously reported that cell lines overexpressing P-gp exhibited reduced sensitivity to IO compared with those cell lines lacking P-gp expression [42]. Upregulation of P-gp was regarded as a mechanism of IO resistance consistent with previous observations of GO resistance and is biologically plausible [29, 30] but had not been investigated in cell lines that had acquired IO resistance. The present study successfully established IO-resistant sublines and demonstrated that this acquired resistance involved the cellular efflux of calicheamicin driven by upregulated P-gp expression. In contrast, another study reported that P-gp expression remained unchanged during IO treatment in clinical settings [42]. Further investigations using samples from ALL patients who develop IO resistance are required to elucidate the clinical significance of P-gp expression. Notably, inhibition of P-gp alone did not fully restore the IC_50_ to the level observed in parental cells, suggesting the involvement of additional resistance mechanisms [43].

Our previous study demonstrated that the addition of PARP inhibitors blocked the repair of IO-induced DNA strand breaks, leading to the augmentation of IO cytotoxicity in B-ALL cells [22]. In the present study, the cytotoxicity of IO was enhanced by the addition of PARP inhibitors, similar to that of IO-resistant cells. In the triple combination, DNA strand breaks were observed at 1 h and remained sustained at the 6—h time point. On the other hand, in combination with IO and a P-gp inhibitor, DNA damage was repaired after 6 h (Fig. 6F, G). These results suggest that a P-gp inhibitor prevented the efflux of calicheamicin, leading to the initial DNA damage. A PARP inhibitor then inhibited the repair of DNA damage induced by accumulated calicheamicin, resulting in the augmentation of apoptosis. A combined approach integrating resistance reversal and strategies to enhance efficacy markedly improved the antitumor effects of IO. Although PARP was not directly involved in the mechanism of resistance, PARP inhibition enhanced calicheamicin-induced DNA damage, thereby enhancing treatment efficacy.

While our study provides important insights into the pharmacological overcoming of IO resistance in vitro, several limitations should be acknowledged. Although the combination of IO with a P-gp inhibitor and a PARP inhibitor effectively overcame resistance in IO-resistant cell lines, clinical translation faces practical challenges, including insurance coverage and potential treatment-related toxicities. Furthermore, it remains unclear whether IO-resistant cells exhibit cross-resistance to other therapies for R/R ALL, such as Blinatumomab or CAR-T therapy, both of which target CD19. Further evaluation of CD19 expression in the IO-resistant cell lines will be essential to clarify potential cross-resistance and to better define the clinical relevance of this three-drug combination strategy.

In conclusion, these findings demonstrate that the enhancement of drug efflux through P-gp overexpression is a key mechanism of IO resistance. Simultaneously inhibiting both drug efflux and DNA damage repair may represent a potential therapeutic strategy to overcome IO resistance in ALL.

## Data Availability

All data supporting the findings of this study are available within the paper and its Supplementary Information.

## References

[1] Kantarjian H, Jabbour E. Adult acute lymphoblastic leukemia: 2025 update on diagnosis, therapy, and monitoring. Am J Hematol. 2025;100(7):1205–31. 40377367 10.1002/ajh.27708 PMC12712861

[2] Kantarjian H, Aldoss I, Jabbour E. Management of adult acute lymphoblastic leukemia: a review. JAMA Oncol. 2025;11(7):771–8. 40310617 10.1001/jamaoncol.2025.0613 PMC12529734

[3] Malard F, Mohty M. Acute lymphoblastic leukaemia. The Lancet. 2020;395(10230):1146–62. 10.1016/S0140-6736(19)33018-1 32247396

[4] DeAngelo DJ, Jabbour E, Advani A. Recent advances in managing acute lymphoblastic leukemia. Am Soc Clin Oncol Educ Book. 2020;40:330–42. 32421447 10.1200/EDBK_280175

[5] Inaba H, Mullighan CG. Pediatric acute lymphoblastic leukemia. Haematologica. 2020;105(11):2522–39. 10.3324/haematol.2020.247031 PMC7604619 33054110

[6] Adomako J, Jiménez-Camacho KE, Correa-Lara MVM, Núñez-Enriquez JC, Schnoor M. Acute lymphoblastic leukemia relapse: biomarkers, hopes, and challenges. Trends in Molecular Medicine [Internet]. 2025 May 23 [cited 2025 Jul 21];0(0). Available from: https://www.cell.com/trends/molecular-medicine/abstract/S1471-4914(25)00107-8 10.1016/j.molmed.2025.04.005 40413101

[7] Seiter K. Therapy for relapsed acute lymphoblastic leukemia: still a role for standard chemotherapy regimens? Leuk Res. 2016;41:1–2. 26764223 10.1016/j.leukres.2015.12.004

[8] Moukalled N, Ferhat AT, Bazarbachi A, Blaise D, Broers AEC, Castilla-Llorente C, et al. Improved outcomes over time in patients aged ≥60 years undergoing allogeneic hematopoietic cell transplantation for acute lymphoblastic leukemia in first complete remission: a large study by the EBMT Acute Leukemia Working Party. Bone Marrow Transplant. 2025;28:1–9. 10.1038/s41409-025-02630-1 40437045

[9] Marcoux C, Kebriaei P. SOHO State of the Art Updates and Next Questions | Transplant in adult acute lymphoblastic leukemia. Clinical lymphoma, myeloma and leukemia [Internet]. 2025 Apr 22 [cited 2025 Jul 22];0(0). Available from: https://www.clinical-lymphoma-myeloma-leukemia.com/article/S2152-2650(25)00147-8/abstract 10.1016/j.clml.2025.04.016 40383653

[10] Wilke AC, Gökbuget N. The role of blinatumomab in adult acute B lymphoblastic leukaemia. Br J Haematol. 2025;207(1):27–42. 40368871 10.1111/bjh.20134 PMC12234269

[11] Kantarjian H, Jain N, Litzow MR, Luger SM, Papayannidis C, Ribera JM, et al. The evolving therapeutic revolution in adult acute lymphoblastic leukemia. Cancer. 2025;131(10):e35872. 40323723 10.1002/cncr.35872 PMC12309614

[12] Bruzzese A, Martino EA, Labanca C, Caridà G, Mendicino F, Lucia E, et al. Therapeutic strategies for relapsed or refractory B-cell acute lymphoblastic leukemia in adult patients: optimizing the use of monoclonal antibodies. Eur J Haematol. 2025;114(6):938–52. 40045912 10.1111/ejh.14405 PMC12053959

[13] McNerney KO, Schultz LM. Tisagenlecleucel in practice: real-world lessons in pediatric and young adult B-ALL. Transplant Cell Ther. 2025;31(6):351.e1-351.e11. 39993597 10.1016/j.jtct.2025.02.016

[14] Sucre O, Pamulapati S, Muzammil Z, Bitran J. Advances in therapy of adult patients with acute lymphoblastic leukemia. Cells. 2025;14(5):371. 40072099 10.3390/cells14050371 PMC11898990

[15] Csizmar CM, Litzow MR, Saliba AN. Antibody-based and other novel agents in adult B-cell acute lymphoblastic leukemia. Cancers. 2025;17(5):779. 40075627 10.3390/cancers17050779 PMC11899621

[16] Lamb YN. Inotuzumab ozogamicin: first global approval. Drugs. 2017;77(14):1603–10. 28819740 10.1007/s40265-017-0802-5

[17] Dahl J, Marx K, Jabbour E. Inotuzumab ozogamicin in the treatment of acute lymphoblastic leukemia. Expert Rev Hematol. 2016;9(4):329–34. 26783163 10.1586/17474086.2016.1143771

[18] Kantarjian HM, Boissel N, Papayannidis C, Luskin MR, Stelljes M, Advani AS, et al. Inotuzumab ozogamicin in adult acute lymphoblastic leukemia: development, current status, and future directions. Cancer. 2022;130(21):3631–46. 10.1002/cncr.35505 PMC12533514 39093036

[19] Raponi S, Stefania De Propris M, Intoppa S, Laura Milani M, Vitale A, Elia L, et al. Flow cytometric study of potential target antigens (CD19, CD20, CD22, CD33) for antibody-based immunotherapy in acute lymphoblastic leukemia: analysis of 552 cases. Leuk Lymphoma. 2011 Jun 1;52(6):1098–107. 10.3109/10428194.2011.559668 21348573

[20] de Vries JF, Zwaan CM, De Bie M, Voerman JSA, den Boer ML, van Dongen JJM, et al. The novel calicheamicin-conjugated CD22 antibody inotuzumab ozogamicin (CMC-544) effectively kills primary pediatric acute lymphoblastic leukemia cells. Leukemia. 2012;26(2):255–64. 21869836 10.1038/leu.2011.206

[21] Kantarjian HM, DeAngelo DJ, Stelljes M, Martinelli G, Liedtke M, Stock W, et al. Inotuzumab ozogamicin versus standard therapy for acute lymphoblastic leukemia. N Engl J Med. 2016;375(8):740–53. 27292104 10.1056/NEJMoa1509277 PMC5594743

[22] Ida N, Okura M, Tanaka S, Hosono N, Yamauchi T. Combining inotuzumab ozogamicin with PARP inhibitors olaparib and talazoparib exerts synergistic cytotoxicity in acute lymphoblastic leukemia by inhibiting DNA strand break repair. Oncol Rep. 2022;52(1):1–10. 10.3892/or.2024.8749 38785163

[23] Anne Underwood P, Bean PA. Hazards of the limiting-dilution method of cloning hybridomas. J Immunol Methods. 1988;107(1):119–28. 3343515 10.1016/0022-1759(88)90017-8

[24] Vasan N, Baselga J, Hyman DM. A view on drug resistance in cancer. Nature. 2019;575(7782):299–309. 31723286 10.1038/s41586-019-1730-1 PMC8008476

[25] Nussinov R, Tsai CJ, Jang H. Anticancer drug resistance: an update and perspective. Drug Resist Updates. 2021;1(59):100796. 10.1016/j.drup.2021.100796 PMC8810687 34953682

[26] Bukowski K, Kciuk M, Kontek R. Mechanisms of multidrug resistance in cancer chemotherapy. Int J Mol Sci. 2020;21(9):3233. 32370233 10.3390/ijms21093233 PMC7247559

[27] Fenton C, Perry CM. Gemtuzumab ozogamicin. Drugs. 2005;65(16):2205–27. 10.2165/00003495-200565160-00014 16266206

[28] Collados-Ros A, Muro M, Legaz I. Gemtuzumab ozogamicin in acute myeloid leukemia: efficacy, toxicity, and resistance mechanisms—a systematic review. Biomedicines. 2022;12(1):208. 10.3390/biomedicines12010208 PMC10813452 38255313

[29] Yamauchi T, Matsuda Y, Tasaki T, Negoro E, Ikegaya S, Takagi K, et al. Induction of DNA strand breaks is critical to predict the cytotoxicity of gemtuzumab ozogamicin against leukemic cells. Cancer Sci. 2012;103(9):1722–9. 22632031 10.1111/j.1349-7006.2012.02343.x PMC7659370

[30] Yamauchi T, Uzui K, Nishi R, Shigemi H, Ueda T. Gemtuzumab ozogamicin and olaparib exert synergistic cytotoxicity in CD33-positive HL-60 myeloid leukemia cells. Anticancer Res. 2014;34(10):5487–94. 25275045

[31] Inase A, Maimaitili Y, Kimbara S, Mizutani Y, Miyata Y, Ohata S, et al. GSK3 inhibitor enhances gemtuzumab ozogamicin‐induced apoptosis in primary human leukemia cells by overcoming multiple mechanisms of resistance. eJ Haematol. 2022 Dec 122(1):153–64. 10.1002/jha2.600 PMC9928658 36819180

[32] Walter RB, Raden BW, Cronk MR, Bernstein ID, Appelbaum FR, Banker DE. The peripheral benzodiazepine receptor ligand PK11195 overcomes different resistance mechanisms to sensitize AML cells to gemtuzumab ozogamicin. Blood. 2004;103(11):4276–84. 14962898 10.1182/blood-2003-11-3825

[33] Mecklenbrauck R, Heuser M. Resistance to targeted therapies in acute myeloid leukemia. Clin Exp Metastasis. 2023;40(1):33–44. 36318439 10.1007/s10585-022-10189-0 PMC9898349

[34] Nooter K, Stoter G. Molecular mechanisms of multidrug resistance in cancer chemotherapy. Pathol Res Pract. 1996;192(7):768–80. 8880878 10.1016/S0344-0338(96)80099-9

[35] Motoji T, Motomura S, Wang YH. Multidrug resistance of acute leukemia and a strategy to overcome it. Int J Hematol. 2000;72(4):418–22. 11197207

[36] Takeshita A. Efficacy and resistance of gemtuzumab ozogamicin for acute myeloid leukemia. Int J Hematol. 2013;97(6):703–16. 23709007 10.1007/s12185-013-1365-1

[37] Marchetti S, Mazzanti R, Beijnen JH, Schellens JHM. Concise review: clinical relevance of drug-drug and herb drug interactions mediated by the ABC transporter ABCB1 (MDR1, P-glycoprotein). Oncologist. 2007;12(8):927–41. 17766652 10.1634/theoncologist.12-8-927

[38] Marchetti S, Mazzanti R, Beijnen JH, Schellens JHM. Concise review: clinical relevance of drug–drug and herb–drug interactions mediated by the ABC transporter ABCB1 (MDR1, P-glycoprotein). Oncologist. 2007;12(8):927–41. 17766652 10.1634/theoncologist.12-8-927

[39] Zhang L, Strong JM, Qiu W, Lesko LJ, Huang SM. Scientific perspectives on drug transporters and their role in drug interactions. Mol Pharm. 2006;3(1):62–9. 16686370 10.1021/mp050095h

[40] Mizuno N, Sugiyama Y. Drug transporters: their role and importance in the selection and development of new drugs. Drug Metab Pharmacokinet. 2002;17(2):93–108. 15618657 10.2133/dmpk.17.93

[41] Takeshita A, Shinjo K, Yamakage N, Ono T, Hirano I, Matsui H, et al. CMC-544 (inotuzumab ozogamicin) shows less effect on multidrug resistant cells: analyses in cell lines and cells from patients with B-cell chronic lymphocytic leukaemia and lymphoma. Br J Haematol. 2009;146(1):34–43. 19388933 10.1111/j.1365-2141.2009.07701.x

[42] Matsumoto T, Jimi S, Hara S, Takamatsu Y, Suzumiya J, Tamura K. Importance of inducible multidrug resistance 1 expression in HL-60 cells resistant to gemtuzumab ozogamicin. Leuk Lymphoma. 2012;53(7):1399–405. 22242821 10.3109/10428194.2012.656102

[43] Wintering A, Ishiyama K, Tamaki S, Tamaki C, Fandel J, Ji L, et al. CD22low/Bcl-2high expression identifies poor response to inotuzumab ozogamicin in relapsed/refractory acute lymphoblastic leukemia. Blood Adv. 2023;7(2):251–5. 35500285 10.1182/bloodadvances.2021006810 PMC9840233

